# Prognostic significance of expression of cysteine-rich 61 and cyclooxygenase-2 in gastric cancer

**DOI:** 10.1186/s12876-016-0478-4

**Published:** 2016-07-25

**Authors:** Yan Wang, Mengchun Wang

**Affiliations:** Department of Gastroenterology, Shengjing Hospital of China Medical University, Shenyang, 110004 China

**Keywords:** Cysteine-rich 61, Cyclooxygenase-2, Gastric cancer, Prognostic significance

## Abstract

**Background:**

This study aimed to evaluate the clinical significance of cysteine-rich 61 (Cyr-61/CCN1) and cyclooxygenase-2 (COX-2), and further explored their combined prognostic significance in gastric cancer.

**Methods:**

This retrospective study examined the expressions of Cyr-61 and COX-2 in 82 surgically removed gastric cancer specimens and 43 non-tumor gastric mucosa specimens by immunohistochemical staining to identify the abnormal expression of Cyr-61 or COX-2 in gastric cancer. Crude survival curves were constructed by the Kaplan-Meier method and Cox proportional hazards regression analysis was performed to confirm the prognostic roles of Cyr-61/COX-2 as well as sex and histological grade.

**Results:**

The expressions of Cyr-61 (*p* < 0.001) and COX-2 (*p* = 0.001) were both significantly up-regulated in gastric cancer samples compared with non-tumor gastric mucosa samples. The high expression of Cyr-61 or COX-2 was associated with invasion, lymph node metastasis, distant metastases, poor histological differentiation, advanced TNM stage and lower 5-year survival rate (all *p* < 0.05). Both Cyr-61 and COX-2 high expressions [hazard ratio (HR) = 31.8, 95 % confidence interval (CI) 4.09–246.8] was associated the higher risk of death during 5 years follow up than single Cyr-61 high expression (HR = 4.1, 95 % CI 1.5–11.6) or COX-2 high expression (HR = 2.9, 95 % CI 1.06–7.8).

**Conclusions:**

Cyr-61 and COX-2 expressions are associated with the progression of gastric cancer. Additionally, combined expressions of Cyr-61 and COX-2 has a higher prognostic value than single expression.

## Background

Gastric cancer is one of the most prevalent and the second leading cause of malignant diseases worldwide [1]. In spite of the gradually decreased incidence, approximately 700,000 mortalities annually are caused by gastric cancer worldwide [2]. Although the treatments for gastric cancer have been considerably developed, the prognosis of patients with advanced cancer is still poor due to the high rate of metastasis [3]. Therefore, it is still imperative to identify the molecular markers for the prognostic evaluation of gastric cancer.

Cysteine-rich 61 (Cyr-61/CCN1) is a member of the connective tissue growth factor family (CCN), which plays important roles in cell proliferation, adhesion, migration, angiogenesis and apoptosis. Studies in recent years have revealed the link between Cyr-61 expression and multiple malignant tumors such as lung cancer [4], breast cancer [5], pancreatic cancer [6], oral squamous cell carcinoma [7] and glioma [8]. Cyr-61 has also been implicated in the development and progression in gastric cancer [9]. Moreover, Maeta et al. reported that Cyr-61 expression in gastric cancer was significantly related to tumor stage, histological differentiation, depth of tumor invasion, lymphatic and venous invasion, and lymph node metastasis [10]. However, the prognostic significance of Cyr-61 in gastric cancer has not been fully determined.

Recent studies have shown that COX-2 highly expressed in esophageal cancer [11], colorectal cancer [12] and other malignant tumors of the digestive tract such as gastric cancer [13]. A recent meta-analysis revealed that high COX-2 expression was associated with an unfavorable overall survival of patients with gastric cancer [14]. Lin et al. [15] further demonstrated that the COX-2 was functionally linked to Cyr-61, and COX-2 played an important role in Cyr-61-promoted invasion and motility in gastric cancer. These studies prompt that Cyr-61 and COX-2 may jointly function in the occurrence and development of gastric cancer, and the prognostic value of the combination of Cyr-61 and COX-2 may higher than that of the single Cyr-61 or COX-2. Thus, in the current study, we evaluated the clinical significance of Cyr-61/COX-2 co-expression in determining the clinicopathologic features of gastric cancer and further explored their combined prognostic significance.

## Methods

### Patients and tissue materials

This retrospective study was approved by the Ethics Committee in the China Medical University, which conforms to the provisions of the World Medical Association’s Declaration of Helsinki in 2013 [16]. Gastric cancer specimens were obtained from 82 patients with primary gastric cancer by gastrectomy at Shengjing Hospital of China Medical University between 2003 and 2005. To identify the abnormal expressions of Cyr-61 and COX-2 in gastric cancer, total 43 non-tumor gastric mucosa specimens were collected as the control by Endoscopy during same period. All patients had not undergone radiotherapy or chemotherapy prior to surgery. The permission to use the tissue materials without informed content was provided by the Ethics Committee of Shengjing hospital (no 2011113). The patients were followed up from the time of surgery until June 2010. Terminal event includes death caused by recurrence and metastasis of gastric cancer. The clinicopathologic records of patients including diameter of tumor, infiltration depth, lymph node metastasis, distance metastasis, histological grade, and tumor node metastasis (TNM) stage were collected.

### Streptavidin-perosidase (S-P) immunohistochemical staining

Immunohistochemical staining of biopsy specimens surgically collected from primary gastric cancer tissues as well as non-tumor gastric mucosa specimens was carried out following manufacturer’s instruction (Fuzhou MaiXin biotechnology Co., Ltd, Fuzhou, China.) [17]. Paraffin sections of tissues were processed and rehydrated in xylene. Slides were incubated in 3 % hydrogen peroxide solution for 10 min to block endogenous peroxidase activity. Antigen retrieval was performed by high temperature and pressure (in EDTA, PH 9.0). Then, slides were blocked by serum, incubated with 1:200 diluted anti-Cyr-61 antibody (Abcam, UK) or 1:100 diluted anti-COX-2 antibody (Boaosen, China) at 4 °C overnight. Phosphate Buffer solution was used to replace the primary antibody in the negative controls. After further incubation with appropriate secondary antibody followed by the incubation with streptavidin-perosidase method, slides were stained with diaminobenzidine (DAB) and hematoxylin, dehydrated and sealed.

Positive staining of Cyr-61 and COX-2 was determined by the detection of brown particles in the cytoplasm under a binocular light microscope (Nikon, Eclipse E100). Staining results were divided into the following category based on percentage of positive cells in total cells: < 25 % as low expression and ≥ 25 % as high expression [18]. Based on this category, the patients with gastric cancer were divided into Cyr-61 or COX-2 high and low expression groups, in order to explore the relationship of these two proteins with clinicopathologic features of gastric cancer as well as evaluate the prognostic value of these two proteins. Six specimens were assessed for each patient with a homogeneous staining.

### Statistical analysis

Data analysis was performed by using SPSS 17.0 software (SPSS Inc., Chicago, IL, USA). Expression of Cyr-61 and COX-2 between gastric carcinoma tissues and non-tumor tissues as well as clinicopathologic features between low and high expressions of Cyr-61/COX-2 groups were compared by Fisher test. Crude survival curves were constructed by the Kaplan-Meier method and survival rates between low and high expression of Cyr-61/COX-2 groups were compared by the log-rank test. To confirm the prognostic roles of Cyr-61 and COX-2, cox proportional hazards regression analysis with a enter method was used to calculate the hazard ratio (HR) with adjustment by sex and histological grade in multivariate analysis. *P* values of less than 0.05 were considered statistically significant.

## Results

### Patient characteristics

A total of 82 cancer patients were enrolled in our study, including 58 (70.73 %) males and 24 (29.27 %) females. The average age of the whole cohort was 55.61 years, with a range from 25 to 79 years. Of the 82 patients, 31 cases were well differentiated and 51 were poorly differentiated. The number of these patients with T1-T4 stage of gastric cancers was 4, 24, 25 and 29, respectively; the number of patients with TNM stage I-IV was 19, 13, 25 and 25, respectively. Fifty-six cases had lymph node metastasis and 23 had distant metastasis. Finally, a total of 55 patients (31 males, 14 females) were completely followed-up. These 55 patients were aged 25–79 years (average age of 67.64 years).

### Cyr-61/COX-2 expression between gastric cancer and non-tumor gastric mucosa

**Fig. 1:**
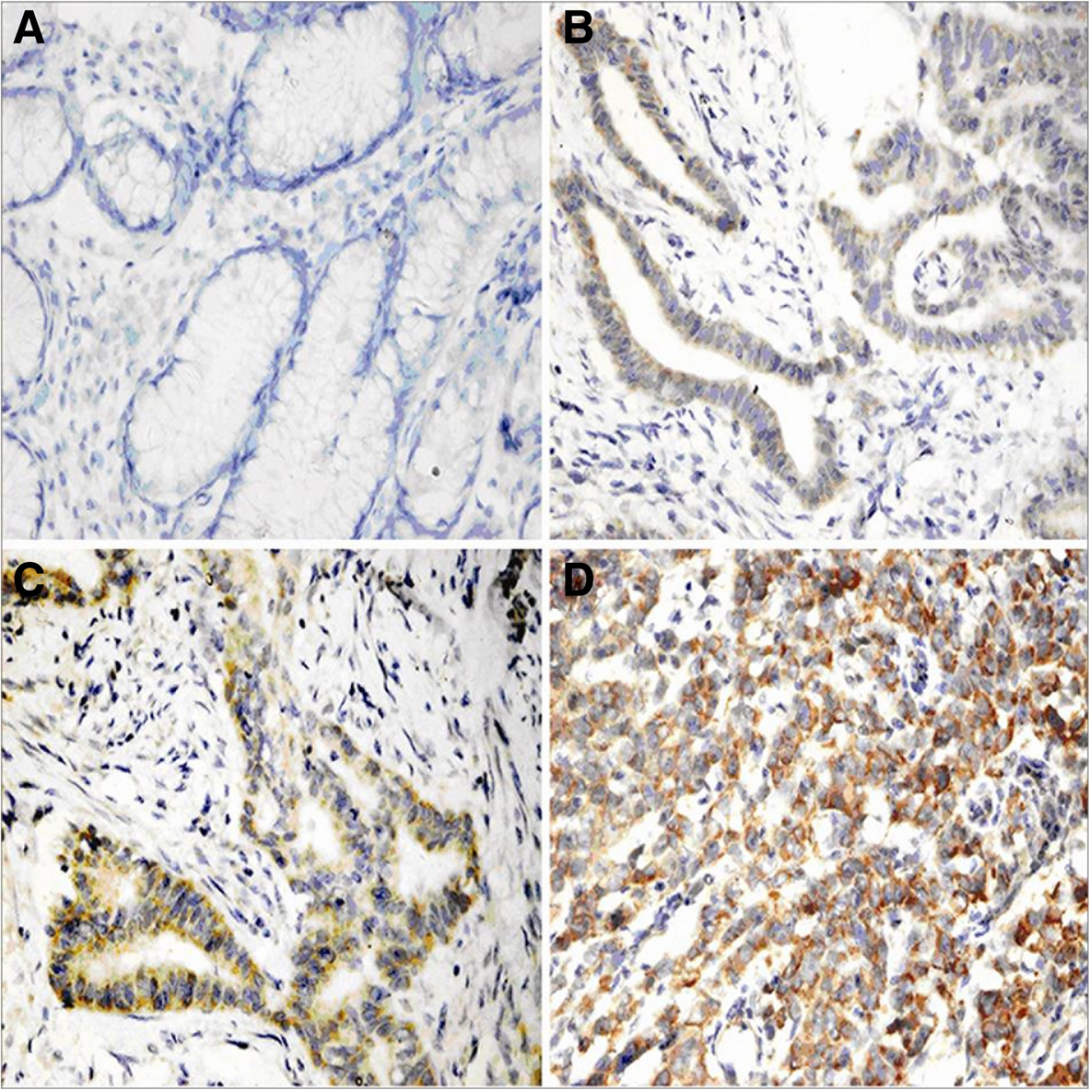
Cyr-61 expression levels in gastric cancer tissues and non-tumor gastric mucosa tissue. Positive staining was mainly located in the cytoplasm of the cells. **a**. negative expression in non-tumor mucosa; **b**. positive expression in well-differentiated gastric cancer; **c**. positive expression in moderately differentiated gastric cancer; **d**. strong positive expression in poorly differentiated gastric cancer

**Fig. 2:**
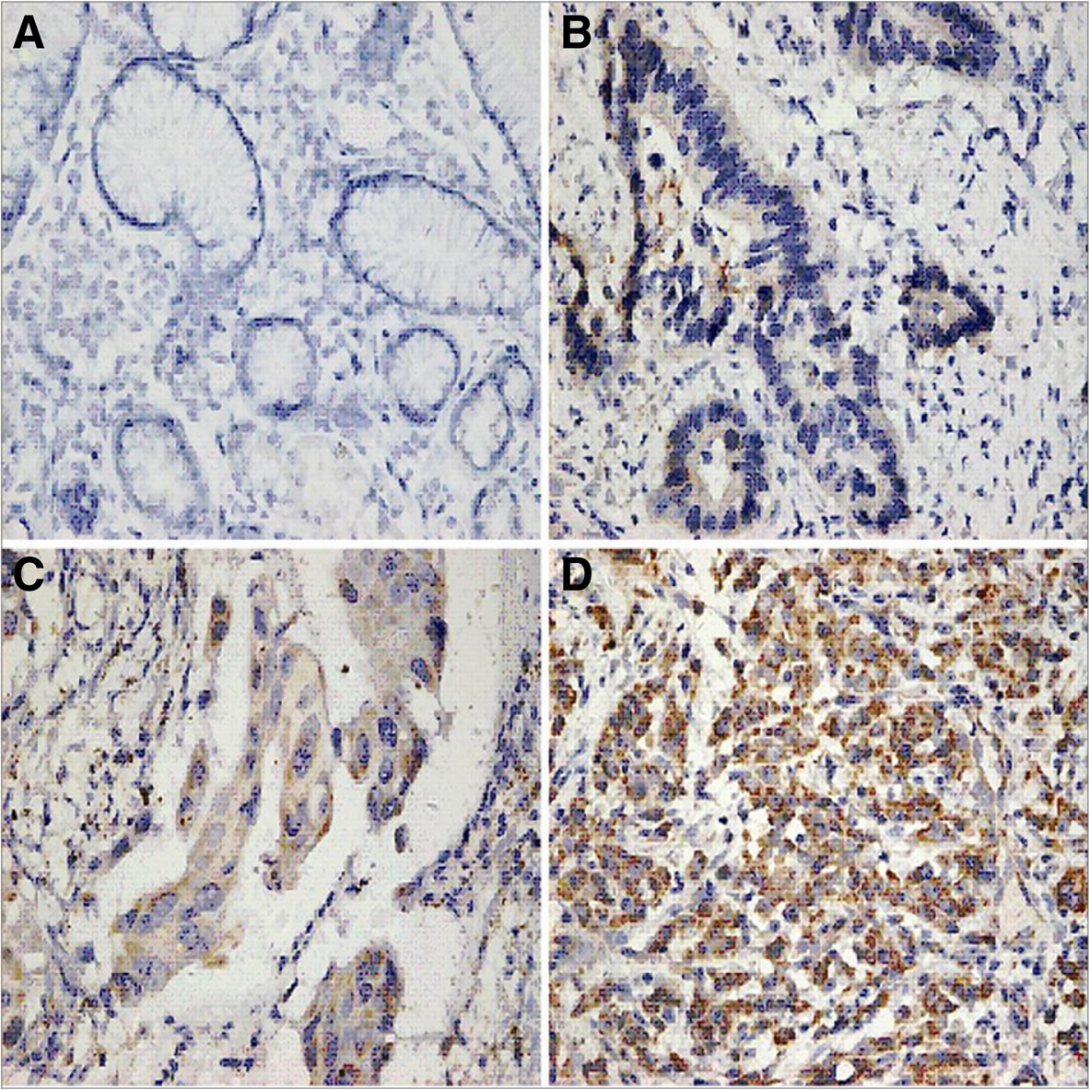
COX-2 expression levels in gastric cancer group and non-tumor gastric mucosa group. Positive staining was mainly located in the cytoplasm of tumor cells. **a**. negative expression in non-tumor mucosa; **b**. positive expression in well-differentiated gastric cancer; **c**. positive expression in moderately differentiated gastric cancer; **d**. strong positive expression in poorly differentiated gastric cancer

Immunohistochemical staining revealed that Cyr-61 expression levels in gastric cancer tissues was increased compared with non-tumor gastric mucosa tissues, and which were enhanced with the decrease of the degree of gastric cancer differentiation (Fig. 1). Among the total 82 gastric cancer samples, 50 (60.98 %) exhibited high expression of Cyr-61 (Table 1). By contrast, only 34.88 % (15/43) of non-tumor gastric mucosa group displayed high expression of Cyr-61 (Table 1). As shown in Table 1, the difference in expression of Cyr-61 between gastric cancer and non-tumor gastric mucosal tissues was significant (*p* = 0.008). In addition, COX-2 expression levels had a similar trend with the expression of Cyr-61 (Fig. 2). Fifty-one (62.2 %) gastric cancer tissues showed high expression of COX-2, while only 18 (41.86 %) of non-tumor gastric mucosa tissues displayed high expression of COX-2 (Table 1). There was significant difference in COX-2 expression between gastric cancer and non-tumor gastric mucosa specimens (*p* < 0.038) (Table 1). A total of 37 out of 82 gastric cancer tissues showed high expressions of both Cyr-61 and COX-2, while 18 exhibited both low expressions, with a coincidence rate of 67.07 % [(37 + 18)/82].

**Table 1:** Expression of Cyr-61 and COX-2 in gastric carcinoma a and non-tumor tissues

Group	Gastric cancer	Non-tumor gastric mucosa	*p*
Cyr-61			0.008
High expression	50 (60.98 %)	15 (34.88 %)	
Low expression	32 (39.02 %)	28 (65.12 %)	
COX-2			0.038
High expression	51 (62.20 %)	18 (41.86 %)	
Low expression	31 (37.80 %)	25 (58.14 %)	

Abbreviations: *Cyr-61* cysteine-rich 61, *COX-2* cyclooxygenase-2

### Cyr-61/COX-2 expression level and clinical characteristics of patients with gastric cancer

Further analyses found that the expression of Cyr-61 was closely associated with invasion depth (*p* = 0.027), lymph node metastasis (*p* = 0.001), distant metastases (*p* = 0.045), poor histological differentiation (*p* = 0.031), and advanced TNM staging (*p* = 0.009). However, the expression level of Cyr-61 was not related to the diameter of tumor (*p* = 0.190), or gender (*p* = 0.752) (Table 2). In addition, COX-2 expression was significantly related to the diameter of tumor (*p* = 0.003), invasion depth (*p* < 0.001), lymph node metastasis (*p* < 0.001), distant metastases (*p* = 0.017), poor tissue differentiation (*p* = 0.015), and advanced TNM staging (*p* < 0.001), but not the gender of patients (*p* = 0.971) (Table 2).

**Table 2:** Relationship between Cyr-61/COX-2 protein levels and clinicopathologic features of patients with gastric cancer

Characteristic	Number	Cyr-61			COX-2		
		High	Low	*p*	High	Low	*p*
Diameter of tumor				0.190			0.003
≥4	58	38	20		42	16	
<4	24	12	12		9	15	
Infiltration depth				0.027			<0.001
T1	4	1	3		1	3	
T2	24	10	14		7	17	
T3	25	17	8		20	5	
T4	29	22	7		23	6	
Lymph node metastasis				0.001			< 0.001
Yes	56	41	15		42	14	
No	26	9	17		9	17	
Distance metastasis				0.045			0.017
Yes	23	18	5		19	4	
No	59	32	27		32	27	
Histological grade				0.031			0.015
I	17	6	11		7	10	
II	22	13	9		11	11	
III	43	31	12		33	10	
Gender				0.752			0.971
Male	58	36	22		36	22	
Female	24	14	10		15	9	
TNM stage				0.009			< 0.001
I	19	6	13		3	16	
II	13	7	6		8	5	
III	25	17	8		20	5	
IV	25	20	5		20	5	

Abbreviations: *Cyr-61* cysteine-rich 61, *COX-2* cyclooxygenase-2, *TNM* tumour node metastasis

### Prognostic role of Cyr-61/COX-2 expression in survival of gastric cancer patients

**Fig. 3:**
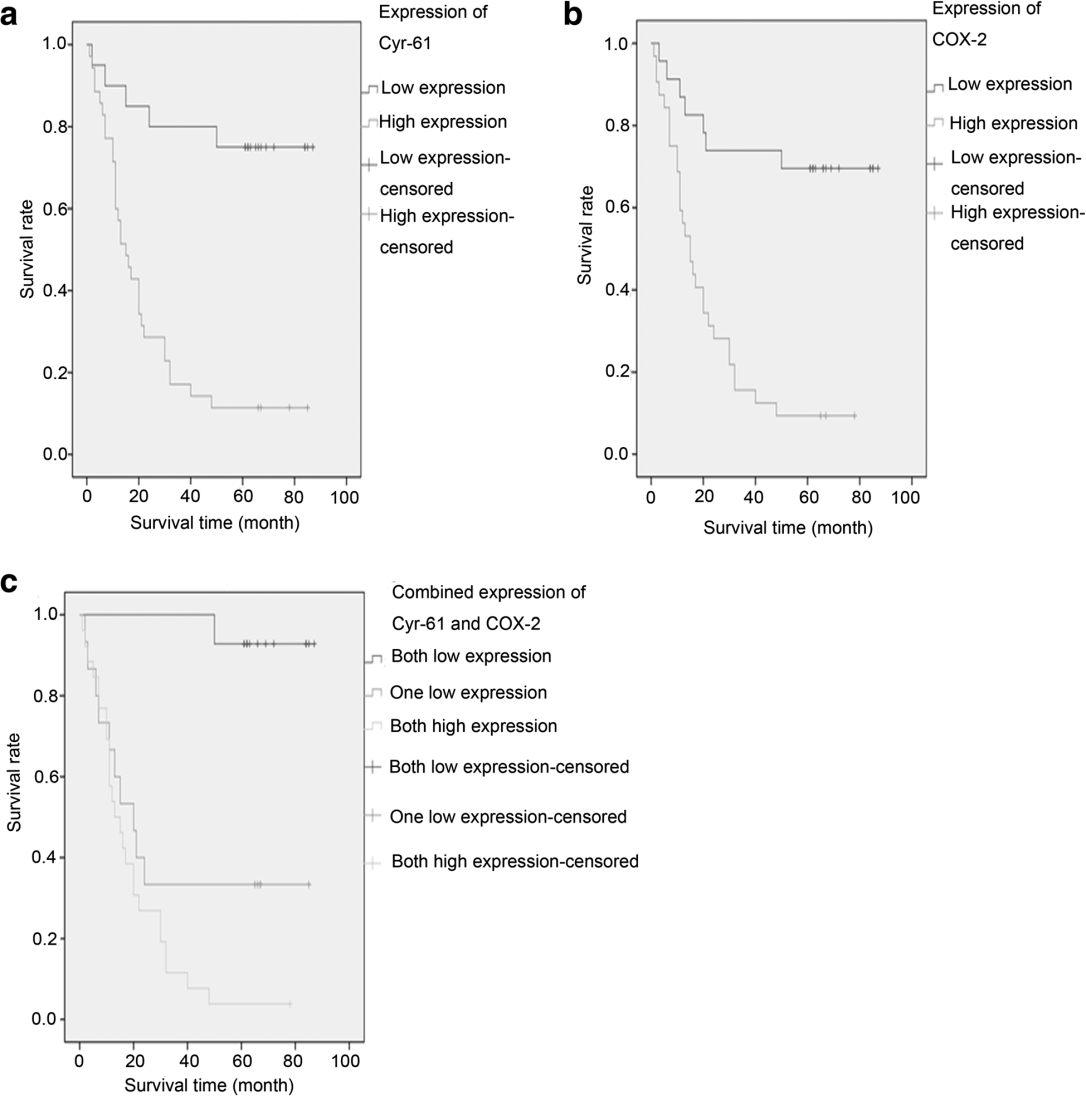
Kaplan-Meier curves for survival analyses. **a**, Kaplan-Meier curves for cum survivals of Cyr-61 expression. **b**, Kaplan-Meier curves for cum survivals of COX-2 expression. **c**, Kaplan-Meier curves for cum survivals of combined expression of Cyr-61 and COX-2

Of the whole cohort, the 5-year survival rate of patients with high Cyr-61 expression was 11.4 %, while that of the patients with low Cyr-61 expression was 75.0 %. The 5-year survival rate of patients with high levels of COX-2 expression was 9.4 %, while the survival rate of the patients with low COX-2 expression was 69.60 %. In addition, the 5-year survival rate of patients with high expressions of both Cyr-61 and COX-2 was 3.8 %, while that of the patients with low expressions of both Cyr-61 and COX-2 was 92.3 %. Furthermore, Kaplan–Meier curves of the Cyr-61 or COX-2 high expression and low expression are shown in Fig. 3a, b, with *p* < 0.001 consistently, demonstrating that the expression of Cyr-61 or COX-2 was associated with the survival time of gastric cancer patients. Also, Kaplan–Meier curve stratified by the combined expressions of Cyr-61 and COX-2 demonstrated high expressions of both Cyr-61 and COX-2 showed the shortest survival time of gastric cancer patients, while low expressions of both Cyr-61 and COX-2 showed the longest survival time of gastric cancer patients (*p* < 0.001, Fig. 3c).

To confirm the prognostic role of Cyr-61 or COX-2, the Cox proportional hazards regression models were adopted. Meanwhile, the other factors (including gender and histological grade) were also analyzed for exploring additional prognostic factors. The univariate analyses demonstrated that histological grade (*p* = 0.031), Cyr-61 expression (*p* < 0.001), COX-2 expression (*p* < 0.001), and the combined expressions of Cyr-61 and COX-2 (*p* = 0.001) were all significantly associated with the survival of gastric cancer patients.

Further multivariate analyses demonstrated that Cyr-61 expression (*p* = 0.008), COX-2 expression (*p* = 0.039), and the combined expressions of Cyr-61 and COX-2 (*p* = 0.003) were prognostic factors independent of gender and histological grade (Table 3). Moreover, both Cyr-61 and COX-2 high expressions [HR = 31.8 (4.1–246.8)] were associated the higher risk of death during 5 years follow up than Cyr-61 high expression [HR = 4.1 (1.5–11.6)] or COX-2 high expression [HR = 2.9 (1.06–7.8)] only.

**Table 3:** Cox proportional hazard regression model analyses of whole cohort

	Univariate		Multivariate^a^		Multivariate^b^	
	HR (95 % CI)	*p*	HR (95 % CI)	*p*	HR (95 % CI)	*p*
Gender		0.316		0.968		0.704
Male	1		1		1	
Female	0.7 (0.3–1.5)		1.1 (0.4–2.5)		0.8 (0.3–2.07)	
Histological grade		0.031		0.563		0.697
Grade I-II	1		1		1	
Grade III	2.2 (1.08–4.5)		1.2 (0.6–2.6)		1.2 (0.6–2.4)	
Cyr-61 expression		< 0.001		0.008		-
Low	1		1			
High	6.9 (2.6–18.04)		4.1 (1.5–11.6)			
COX-2 expression		< 0.001		0.039		-
Low	1		1			
High	5.6 (2.4–13.03)		2.9 (1.06–7. 8)			
Combined expression of Cyr-61 and COX-2		0.001		-		0.003
Both low	1				1	
One low	18.2 (2.3–142.7)	0.006			17.9 (2.2–143.3)	0.006
Both high	34.8 (4.6–262.4)	0.001			31.8 (4.09–246.8)	0.001

Abbreviation: *HR* hazard ratio, *CI* confidence interval; ^a^multivariate Cox regression analysis including sex, histological grade, Cyr-61 expression and COX-2 expression; ^b^multivariate Cox regression analysis including sex, histological grade, and combined expression of Cyr-61 and COX-2

## Discussion

In the present study, we examined the expression of Cyr-61 and COX-2 in 82 gastric cancer specimens and 43 non-tumor gastric mucosa specimens, and the results demonstrated the clinical significance of the expressions of Cyr-61 and COX-2 in determining the clinicopathologic features of gastric cancer and predicting its prognostic value.

Our results showed that Cyr-61 expression in gastric cancer tissues was significantly higher than that in the non-tumor gastric mucosa tissues. Furthermore, gastric cancer with higher Cyr-61 expression was associated with stronger invasion and metastasis abilities, advanced TNM stage and shorter survival time, suggesting that Cyr-61 plays an important role in the progression of gastric cancer. These results were consistent with the findings of Lin et al. [15]. They found Cyr-61 expression level was closely related to stages of gastric cancers, lymph node metastasis, and histological type. The 5-year survival time of patients with high Cyr-61 expression was significantly shorter than that of patients with low Cyr-61 expression [15]. Their further study also showed that cell lines had higher level of Cyr-61 expression, accompanied by significantly higher adhesion ability than those showed lower Cyr-61 expression level [19].

Epidemiological studies, clinical and animal trials all suggested that selective inhibition of COX-2 expression could reduce the incidence of colorectal cancer [20, 21]. Leung et al. [22] found that there was a link between mutations in tumor suppressor gene p53 and COX-2 expression in gastric cancers. Specifically, COX-2 expression was significantly increased in gastric cancer tissues with p53 mutation. Coincidently, higher rate of lymph node metastasis and lower survival rate were evidenced in gastric cancer with p53 mutation and high COX-2 expression. In addition, Min et al. [23] found that COX-2 expression in gastric cancers was positively correlated with the expression of oncogene K-ras, while both gene expressions were correlated with the depth of tumor invasion and rate of lymphatic metastasis. Our results showed that COX-2 expression in gastric cancers was significantly different from that in the non-tumor gastric mucosa. In addition, COX-2 expression was significantly associated with tumor size, depth of invasion, rate of lymph node metastasis, distant metastasis, and pathological stage of the tumor. Furthermore, patients with high COX-2 expression revealed decreased survival time.

Cyr-61 had been reported to mediate extensive cellular processes, including cell adhesion, cell motility, cell invasion, cell survival, and cell proliferation [24–26]. Lin et al. [19] suggested that Cyr-61 might promote the adhesion ability of gastric tumor cells through up-regulating the functional integrin α2β1 via an AP-1-dependent signaling pathway, therefore, contribute to the peritoneal dissemination of gastric cancer [19]. Furthermore, Lin et al. [27] found that Cyr-61 could enhance gastric cancer cell invasion by promoting the expression of hypoxia-inducible factor-1 (HIF-1). Interestingly, COX-2, an important rate-limiting enzyme in prostaglandin synthesis, had been demonstrated to promote angiogenesis by increasing the expression of HIF-1 in gastric carcinoma [28]. It had been confirmed that COX-2 was closely associated with gastric carcinoma progression through regulating tumor proliferation, apoptosis, invasiveness and angiogenesis [29, 30]. Through clinical trials and in vitro experiments, Lin et al. [15] further confirmed that *COX-2* was a downstream gene induced by Cyr-61, and Cyr-61 could affect the invasion ability of gastric cancer cells by upregulating COX-2 activity via the αVβ3/NF-kB pathway. These results indicated that the Cyr-61 and COX-2 might have a synergistic promoting effect on the progression of gastric carcinoma. Therefore, we speculated that the higher prognostic value of combined expression of Cyr-61 and COX-2 than Cyr-61 or COX-2 alone might be caused by the synergistic mechanism in the progression of gastric cancer.

Undoubtedly, our study has several limitations. The first limitation is the small sample and the retrospective nature of this study, more prospective studies with larger sample size should be performed to confirm the results of this study. Second, because the data are retrospectively collected, no available data can be used to confirm the prognostic value of combined expression of Cyr-61 and COX-2 by receiver operating characteristic curve. Third, the current study only compared the Cyr-61/COX-2 protein levels between the GC tissues and non-tumor tissues, while the both detections of COX-2 and Cyr-61 RNA and protein levels may be more comprehensive. In addition, we only chose one technique (immunohistochemistry) in this study; however, the conclusions may be more convincing if we supplemented the data of Western blot or in situ hybridization. Fourth, because immunohistochemical figures cannot be exactly distinguish cell types, we did not describe the cell types of COX2 and Cyr-61expressions. Lastly, there is no mechanism study in this study, further investigations for the downstream signaling pathways of Cyr-61/COX-2 and their interaction in gastric cancer are necessary.

## Conclusions

In conclusion, both Cyr-61 and COX-2 expression are associated with the progression of gastric cancer. Meanwhile, prognostic role of Cyr-61 and COX-2 is confirmed in this study. Moreover, combined expression of Cyr-61 and COX-2 has higher prognostic value than single expression for the survival of gastric cancer patients.

## Abbreviations

CI, confidence interval; COX-2, cyclooxygenase-2; Cyr-61/CCN1, cysteine-rich 61; DAB, diaminobenzidine; HR, hazard ratio; TNM, tumor node metastasis

## References

[1] Siegel R, Naishadham D, Jemal A (2012). Cancer statistics, 2012. CA Cancer J Clin.

[2] Kamangar F, Dores GM, Anderson WF (2006). Patterns of cancer incidence, mortality, and prevalence across five continents: defining priorities to reduce cancer disparities in different geographic regions of the world. J Clin Oncol.

[3] Shah MA, Ajani JA (2010). Gastric cancer—an enigmatic and heterogeneous disease. Jama.

[4] Lv J, Zou Y, Zhang C, Mao Z (2010). Expressions of Cyr61 and WISP-3 in non-small cell lung cancer and its clinical significance. Zhongguo Fei Ai Za Zhi.

[5] Nguyen N, Kuliopulos A, Graham RA, Covic L (2006). Tumor-derived Cyr61(CCN1) promotes stromal matrix metalloproteinase-1 production and protease-activated receptor 1-dependent migration of breast cancer cells. Cancer Res.

[6] Holloway SE, Beck AW, Girard L, Jaber MR, Barnett CC, Brekken RA, Fleming JB (2005). Increased expression of Cyr61 (CCN1) identified in peritoneal metastases from human pancreatic cancer. J Am Coll Surg.

[7] Kok SH, Chang HH, Tsai JY, Hung HC, Lin CY, Chiang CP, Liu CM, Kuo MYP (2010). Expression of Cyr61 (CCN1) in human oral squamous cell carcinoma: an independent marker for poor prognosis. Head Neck.

[8] Xie D, Yin D, Tong X, O’Kelly J, Mori A, Miller C, Black K, Gui D, Said JW, Koeffler HP (2004). Cyr61 is overexpressed in gliomas and involved in integrin-linked kinase-mediated Akt and beta-catenin-TCF/Lef signaling pathways. Cancer Res.

[9] Cheng TY, Wu MS, Hua KT, Kuo ML, Lin MT (2014). Cyr61/CTGF/Nov family proteins in gastric carcinogenesis. World J Gastroenterol.

[10] Maeta N, Osaki M, Shomori K, Inaba A, Kidani K, Ikeguchi M, Ito H (2007). CYR61 downregulation correlates with tumor progression by promoting MMP-7 expression in human gastric carcinoma. Oncology.

[11] Huang JX, Xiao W, Chen WC, Lin MS, Song ZX, Chen P, Zhang YL, Li FY, Qian RY, Salminen E (2010). Relationship between COX-2 and cell cycle-regulatory proteins in patients with esophageal squamous cell carcinoma. World J Gastroenterol.

[12] Wu BW, Li DF, Ke ZF, Ma D, Li YJ, Gang D, Zheng ZG, Zhang KJ, Zhang YH (2010). Expression characteristics of heparanase in colon carcinoma and its close relationship with cyclooxygenase-2 and angiogenesis. Hepato-Gastroenterology.

[13] Koga T, Shibahara K, Kabashima A, Sumiyoshi Y, Kimura Y, Takahashi I, Kakeji Y, Maehara Y (2003). Overexpression of cyclooxygenase-2 and tumor angiogenesis in human gastric cancer. Hepato-Gastroenterology.

[14] Song J, Su H, Zhou YY, Guo LL (2014). Cyclooxygenase-2 expression is associated with poor overall survival of patients with gastric cancer: a meta-analysis. Dig Dis Sci.

[15] Lin MT, Zuon CY, Chang CC, Chen ST, Chen CP, Lin BR, Wang MY, Jeng YM, Chang KJ, Lee PH (2005). Cyr61 induces gastric cancer cell motility/invasion via activation of the integrin/nuclear factor-kappaB/cyclooxygenase-2 signaling pathway. Clin Cancer Res.

[16] Association WM (2013). World Medical Association Declaration of Helsinki: ethical principles for medical research involving human subjects. Jama.

[17] Zhang W, Tong Q, Li S, Wang X, Wang Q (2008). MG-132 inhibits telomerase activity, induces apoptosis and G1 arrest associated with upregulated p27kip1 expression and downregulated survivin expression in gastric carcinoma cells. Cancer Investig.

[18] Ishikawa M, Kitayama J, Nariko H, Kohno K, Nagawa H (2004). The expression pattern of UDP‐N‐acetyl‐α‐D‐galactosamine: polypeptide N‐acetylgalactosaminyl transferase‐3 in early gastric carcinoma. J Surg Oncol.

[19] Lin MT, Chang CC, Lin BR, Yang HY, Chu CY, Wu MH, Kuo ML (2007). Elevated expression of Cyr61 enhances peritoneal dissemination of gastric cancer cells through integrin alpha2beta1. J Biol Chem.

[20] Chan AT, Giovannucci EL, Meyerhardt JA, Schernhammer ES, Wu K, Fuchs CS (2008). Aspirin dose and duration of use and risk of colorectal cancer in men. Gastroenterology.

[21] Levy GN (1997). Prostaglandin H synthases, nonsteroidal anti-inflammatory drugs, and colon cancer. FASEB J.

[22] Leung WK, To KF, Ng YP, Lee TL, Lau JY, Chan FK, Ng EK, Chung SC, Sung JJ (2001). Association between cyclo-oxygenase-2 overexpression and missense p53 mutations in gastric cancer. Br J Cancer.

[23] Li M, Liu W, Zhu YF, Chen YL, Zhang BZ, Wang R (2006). Correlation of COX-2 and K-ras expression to clinical outcome in gastric cancer. Acta Oncol.

[24] Grzeszkiewicz TM, Lindner V, Chen N, Lam SC, Lau LF (2002). The angiogenic factor cysteine-rich 61 (CYR61, CCN1) supports vascular smooth muscle cell adhesion and stimulates chemotaxis through integrin alpha(6)beta(1) and cell surface heparan sulfate proteoglycans. Endocrinology.

[25] Babic AM, Kireeva ML, Kolesnikova TV, Lau LF (1998). CYR61, a product of a growth factor-inducible immediate early gene, promotes angiogenesis and tumor growth. Proc Natl Acad Sci U S A.

[26] Lin MT, Chang CC, Chen ST, Chang HL, Su JL, Chau YP, Kuo ML (2004). Cyr61 expression confers resistance to apoptosis in breast cancer MCF-7 cells by a mechanism of NF-kappaB-dependent XIAP up-regulation. J Biol Chem.

[27] Lin M-T, Kuo I-H, Chang C-C, Chu C-Y, Chen H-Y, Lin B-R, Sureshbabu M, Shih H-J, Kuo M-L (2008). Involvement of hypoxia-inducing factor-1α-dependent plasminogen activator inhibitor-1 up-regulation in Cyr61/CCN1-induced gastric cancer cell invasion. J Biol Chem.

[28] Huang S-P, Wu M-S, Shun C-T, Wang H-P, Hsieh C-Y, Kuo M-L, Lin J-T (2005). Cyclooxygenase-2 increases hypoxia-inducible factor-1 and vascular endothelial growth factor to promote angiogenesis in gastric carcinoma. J Biomed Sci.

[29] Wu WKK, Sung JJY, Lee CW, Yu J, Cho CH (2010). Cyclooxygenase-2 in tumorigenesis of gastrointestinal cancers: an update on the molecular mechanisms. Cancer Lett.

[30] Fujimura T, Ohta T, Oyama K, Miyashita T, Miwa K (2006). Role of cyclooxygenase-2 in the carcinogenesis of gastrointestinal tract cancers: a review and report of personal experience. World J Gastroenterol.

